# Cancer-related cognitive impairment in cancer: Examining portuguese cancer survivors’ acceptability and expectations regarding cognitive telerehabilitation interventions

**DOI:** 10.1192/j.eurpsy.2021.1161

**Published:** 2021-08-13

**Authors:** A.F. Oliveira, A. Torres, A. Pereira, I.M. Santos

**Affiliations:** 1 Department Of Education And Psychology Of University Of Aveiro, CINTESIS - UA - Center for Health Technology and Services Research, Portugal (R&D Unit ref. UIDB/4255/2020), Aveiro, Portugal; 2 Nursing, Portuguese Red Cross Northern Health School, Oliveira de Azeméis, Portugal; 3 Department Of Education And Psychology Of University Of Aveiro, Research Centre in Didactics and Technology in the Education of Trainers (CIDTFF), Aveiro, Portugal; 4 Department Of Education And Psychology Of University Of Aveiro, William James Center for Research, Aveiro, Portugal

**Keywords:** Cancer-related cognitive impairment, Portuguese cancer survivors, Acceptability and expectations, cognitive telerehabilitation interventions

## Abstract

**Introduction:**

Cancer-related cognitive impairment (CRCI) is one of the most frequent and worrying side effects experienced by non-central nervous system (CNS) cancer survivors, generally related to cancer treatments. Considering its detrimental impact on quality of life, including work-related outcomes, it is necessary to identify effective intervention options. Cognitive rehabilitation is considered the first-line intervention to address CRCI, being effective at improving cognitive functioning. Internet-based interventions are emerging as important means of intervention in the field of cognitive rehabilitation (known as cognitive telerehabilitation), considering the potential to overcome accessibility issues and being cost-effective.

**Objectives:**

To assess the acceptability and expectations regarding such interventions, considering the scarce literature.

**Methods:**

A nationwide online survey was disseminated to Portuguese non-CNS cancer survivors, aged 18-65 years, who had finished active treatments, with no metastases/history of neurological or psychiatric disease/alcohol or drug abuse. Preexisting knowledge about CRCI, expectations for support to cognitive difficulties, Internet use for health and support purposes, and intervention needs and preferences in the context of cognitive telerehabilitation were examined; sociodemographic and clinical variables (e.g., age, education, employment status, cancer treatments), as well as cognitive complaints (Portuguese version of the Functional Assessment of Cancer Therapy-Cognitive, FACT-Cog), were also assessed.

**Results:**

Findings from this study are important to help health professionals and researchers understand and identify cancer survivors’ needs regarding cognitive telerehabilitation interventions.

**Conclusions:**

This information could be used as a support and guide for the development and delivery of these interventions for non-CNS cancer survivors.

